# Effect of Compound Fields of Ultrasonic Vibration and Applied Pressure on the 3D Microstructure and Tensile Properties of Recycled Al-Cu-Mn-Fe-Si Alloys

**DOI:** 10.3390/ma12233904

**Published:** 2019-11-26

**Authors:** Yuliang Zhao, Bo Lin, Dongfu Song, Donghai Zheng, Zhenzhong Sun, Chunxiao Xie, Weiwen Zhang

**Affiliations:** 1 Neutron Scattering Technical Engineering Research Center, School of Mechanical Engineering, Dongguan University of Technology, Dongguan 523808, China; dhzheng@foxmail.com (D.Z.); sunzz@dgut.edu.cn (Z.S.); xiechx@dgut.edu.cn (C.X.); 2 National Engineering Research Centre of Near-net-shape Forming for Metallic Materials, South China University of Technology, Guangzhou 510641, China; songyuren1015@163.com; 3 School of Mechanical Engineering, Guizhou University, Guiyang 550025, China; linbo1234@126.com; 4 Guangdong Institute of Materials and Processing, Guangzhou 510650, China

**Keywords:** ultrasonic vibration, applied pressure, 3D morphology, Fe-rich phase, grain refinement

## Abstract

The effect of compound fields of ultrasonic vibration and applied pressure (UV+AP) on three-dimensional (3D) microstructure and tensile properties of recycled Al-Cu-Mn-Fe-Si alloys was systematically studied using conventional two-dimensional (2D) microscopy, synchrotron X-ray tomography, and tensile test. The properties of UV+AP treated alloys with the pouring temperature of 740, 710 and 680 °C were compared when those alloys achieved after gravity casting. After UV+AP treatment, the alloy with pouring temperature of 710 °C show the smallest grain size. Also, the sizes of Fe-rich phases and Al_2_Cu are greatly reduced and their 3D morphologies are compacted. The mechanical properties of UV+AP treated alloys are relatively higher than those measured for gravity cast equivalents. This improvement can be explained by the synergistic effect of acoustic cavitation, acoustic streaming, and force-feeding, which resulted in the dendrite fragmentation, uniform solute distribution, and microstructural refinement. The Orowan strengthening and solution strengthening were identified as the main strengthening mechanisms.

## 1. Introduction

High-strength aluminum (Al) alloys are an important structural part for light-weighting vehicles and are increasingly used in aircraft and automobiles industry [1]. Aluminum-Copper (Al-Cu) alloys, as a typical high-strength engineering Al alloys, owe their strength to the dispersed distribution of fine precipitates in the alloys. Al-Cu alloys recycling in aircraft and non-aircraft applications has been widely accepted due to sustainability, energy saving, and emissions and wastes reduction [2,3,4]. However, iron (Fe) accumulates in the Al alloys during their life-cycle and forms the hard and brittle Fe-rich intermetallic phases (called Fe-rich phases hereafter) during solidification [4,5]. The plate-like β-Al_7_Cu_2_Fe or Al_3_(FeMn) severely deteriorates the strength, especially elongation, during tensile test [5]. Si is considered as another impurity element in the high-strength Al-Cu alloys and its content in the alloys is usually limited [2,5]. Si exists in the alloy in two different states: Dissolve in the α-Al matrix and precipitated as Si-containing intermetallic phases [2]. According to the present authors [6], Si addition changes the plate-like β-Al_7_Cu_2_Fe and Al_3_(FeMn) to Chinese-script Al_6_(FeMn), and Al_15_(FeMn)_3_(SiCu)_2_ (α-Fe) and accelerates the aging kinetics of precipitates after heat treatment. In the meantime, Mn addition promotes the formation of Chinese-script Fe-rich phases [7].

The application of external fields, i.e., ultrasonic field, electromagnetic field, electric field, in the Al melting during solidification can refine the grain size and enhance the mechanical properties [8,9,10]. Ultrasonic melt processing (USP) has been considered a promising method to refine the microstructure [8]. The main refine mechanisms involve the implosive collapse of cavitation bubbles fragments the dendrites and intermetallic phases, and acoustic streaming ensure the fragments are uniformly distributed in the melting [8,9]. It is well-recognized that the refined and equiaxed grain structures, pore-elimination and homogenous microstructure can be obtained by USP [11,12,13]. Very limited literature has focused on the effect of USP on the intermetallic phase, such as Fe-rich phases. The application of USP on the different processing temperatures of Al-Si-Cu alloys during solidification was reported, and it has been found that the USP effectively refines the Fe-rich phases [14]. An attempt has made on the high Fe-containing Al-Si alloys (Fe content up to 4%), and it was found that the USP led to the enhancement of peritectic transformation of Fe-rich phases and refined the primary Fe-rich phases and eliminated macro-segregation [15]. Squeeze casting is a liquid forging technology under applied pressure [16], which can obtain pore-free and refined microstructure. Recently investigations [17,18,19] have been carried out to understand the effect of applied pressure on the solidified microstructure. The results show that both the primary Al phases and intermetallic phases can be refined, and the volume fraction of the pore is effectively reduced.

Recently, some attempts [20,21,22,23,24] of the compound fields (such as electromagnetic field and ultrasonic vibration, and ultrasonic vibration and squeeze casting) have been carried out to achieve high-strength Al alloys. They both obtained satisfactory experimental results. Their aim was to obtain refined microstructure through an environmentally–friendly and easy method of compound field. Yue et al. [20], Zhang et al. [22] and Tao et al. [23] employed the compound field of magnetic field and ultrasonic vibrations in the solidification of Al or Al-based composite alloys. They both obtained the refined microstructure and enhanced mechanical properties. Moreover, the compound field is helpful in promoting the melt reaction and ensuring the nanoparticles are homogenously distributed in the alloys. Chen et al. [21] used the indirect ultrasonic vibration and Yuan et al. [24] firstly applied the USP in the crucible and then poured it into the mold for squeeze casting. None of them involve the application of ultrasonic vibration and squeeze casting on the Al melting during solidification simultaneously. Our preliminary experimental studies [25,26,27] showed that the compound field of ultrasonic vibration and applied pressure (UV+AP) can obtain much more refined and homogenous microstructures than individual fields. However, the effect of process parameter, such as pouring temperature, on the microstructure is still unclear.

The development of third generation synchrotron X-ray facilities, with high brilliance X-rays, is available for the three-dimensional (3D) rendering the microstructure [28,29]. In recent years, significant effort has been made to explore the effect of USP on 3D morphology of Fe-rich phases [30,31,32,33]. The experimental results indicate that high cooling rate and Sr addition refined the 3D sizes of Fe-rich phases [30]. The interconnectivity of 3D structures of Fe-rich phases in Al-Cu alloys was significantly reduced after heat treatment [31]. At the same time, in-situ studies of the effect of USP on the evolution of dendrites structure and solid-liquid interface in a 2D or 3D manner were clearly shown [32,33].

In this work, the influence of compound fields of UV+AP on the 3D microstructure and tensile properties of recycled Al-Cu-Mn-Fe-Si alloy was investigated using synchrotron X-ray tomography, tensile test and transmission electron microscopy (TEM). The effect of pouring temperature (680, 710 and 740 °C) on the 3D microstructure of the studied alloys was also examined. In addition, the size, number density, and distribution of precipitates in the alloys were examined using TEM. The mechanisms of UV+AP on the microstructure were briefly established.

## 2. Materials, Methods and Experimental

**Figure 1:**
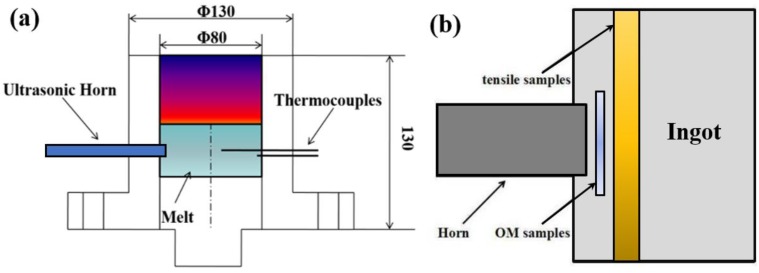
(**a**) Schematic illustration of UV+AP equipment; (**b**) schematic diagram showing optical microscope (OM) observation and tensile test sampling positions.

The design chemical compositions of the studied alloys were Al-5.0Cu-0.6Mn-0.5Fe-0.6Si (weight percentage). The actual chemical compositions were shown in Table 1, which were determined by the optical emission spectrometer. The raw materials were melted at 780 °C in an electric resistance furnace, and then the melting was stirred with C_2_Cl_6_ powder for degassing and slag removal. After that, the melts were holding at the different melting temperatures for 10 min. The schematic diagram of experimental equipment of combined ultrasonic vibration and applied pressure is shown in Figure 1a. The ultrasonic vibration system is consisting of ultrasonic generator (1 kW), magnetostrictive transducer (20-KHz) and titanium ultrasonic sonotrode (10 mm diameter) [34]. The sonotrode was preheated to 200 °C by a resistant heating coil, this step is to reduce the effect of cold sonotrode on the microstructural refinement.

The pouring temperatures were set at approximately 680, 710, and 740 °C, respectively. The aim was to study the degree of melting temperature (20, 50, and 80 °C) above the liquid temperature on the microstructure. The designed ultrasonic power was 900 W with the applied pressure of 50 MPa. They were simultaneously worked on melts for about 30 s, and were then withdrawn at an approximate melt temperature of 545–550 °C. The UV+AP equipment was schematically shown in Figure 1a. The sonotrode tip was directly inserted to the melt with the depth of 10 mm. For the gravity casting alloy, they were also directly contacted the melts for a comparative study. Finally, the solidified samples (Φ80 mm × 68 mm) were obtained under different conditions.

The position of the samples for the microstructure observation and tensile test were cut at the effect zone of the ingots, and are schematically shown in Figure 1b. The samples for conventional 2D observations have a size of Φ10 × 2 mm^2^, and were etched with a 0.5 % hydrofluoric acid solution. For grain characterization, the samples were electro-etching with 20% Fluoroboric acid solution with the voltage of 20 V for 2 min. Then, they were observed in the polarized optical microscope. In particular, the type of the Fe-rich phases and dispersoids at nano- or micro-level were further identified by HRTEM (Tecnai G2 F30 field, Thermo Fisher, Hillsboro, OR, USA) and energy dispersive spectrometer (EDS). The TEM samples were grinded and polished to about 100 μm, and then thinned by ion miller (Gantan 691, Gantan, Pleasanton, CA, USA) to 40–50 μm. The operated voltage for TEM observation was 200 kV. For heat treatment, the samples were solid solution on 538 °C for 12 h and aging at 155 °C for 8 h (Typical T5 heat treatment). Tensile test (SANS CMT5105, Suns, Shenzhen, China) for the as-cast and heat-treatment alloys were performed with applied strain rate of 1 mm/min. The standard rod-like tensile samples were used in this study, the size of central part was 10 mm and the length was 25 mm. At least 3 samples were tested for each condition.

**Figure 2:**
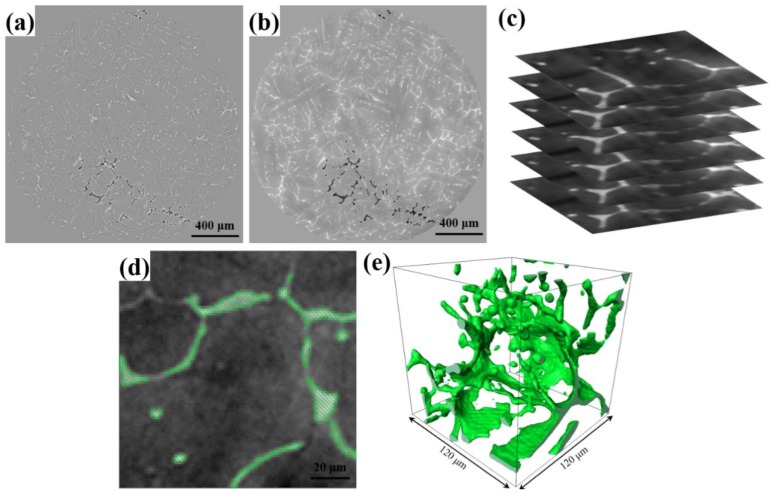
(**a**) A typical two-dimensional (2D) slice from the tomography scan of the gravity casting alloy; (**b**) the image processed using a three-dimensional (3D) bilateral filter; (**c**) the pile-up images in the Avizo software; (**d**) the extracted Al_2_Cu phases according to different threshold values; and (**e**) the 3D volume rendered Al_2_Cu phases.

Synchrotron X-ray tomography experiments were performed at the ID 19 beamline, at the European Synchrotron Radiation Facility (ESRF), in France. The sizes of samples for tomography were Φ2 mm [34,35,36]. A white-beam was used with a microscope of 10× magnification, using a 25 μm LuAG: Ce scintillator and a high-speed CMOS camera (PCO. DIMAX, PCO, Kelheim, Germany). While, 1000 projections with 1.2 μm resolution were recorded over 180° of sample rotation. The image reconstructions were performed on the ESRF cluster using the inhouse developed software-package PyHST_2 (2.0, PyHST, Grenoble, France). Figure 2 shows the typical image processing procedures and methods employed for 3D microstructure reconstruction. We change the contrast between Fe-rich phases, Al_2_Cu and Al matrix using Image J [37] software (1.52a, NIH, Bethesda, USA). Then, all the datasets were used the 3D bilateral filter embedded in the Avizo^®^ Lite v9.0.1 software [38] (VSG, Bordeaux, France) to increase the contrast and reduce noise (Figure 2b). Finally, the Fe-rich phases, Al_2_Cu and Al dendrites were manually trimmed for the different material phases (Figure 2c). All the image segmentation and volume rendering were finished in Avizo^®^ software (Figure 2d). The volume of 240 μm × 240 μm × 240 μm was chosen as the demonstration, the typical 3D morphology of Al_2_Cu was reconstructed (Figure 2e).

The parameter was used to characterize the surface curvature, the mean curvature *H*, [39] is given by,

(1) $$H=0.5*\left(\frac{1}{R_{1}}+\frac{1}{R_{2}}\right)$$

where *R*_1_ and *R*_2_ are the two main curves at the surfaces. The skeletonization function embedded in Avizo software was used to thin the 3D structures of intermetallic phases into 1D line with connecting nodes. Thus, their 3D morphology and size can be characterized by the number of the connecting nodes and node length.

## 3. Results

### 3.1. Optical Microstructure

**Figure 3:**
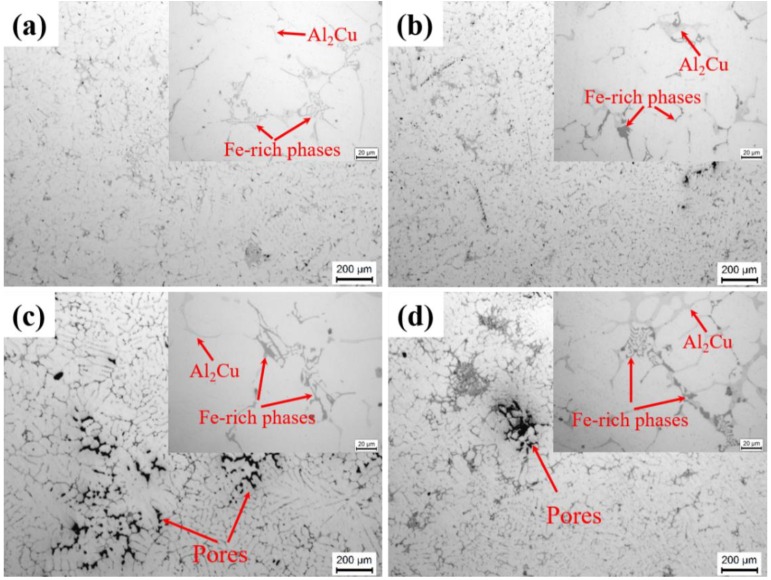
Optical images of the alloys with different pouring temperatures: (**a**) UV+AP, 740 °C; (**b**) UV+AP, 710 °C; (**c**) gravity casting, 710 °C; (**d**) UV+AP, 680 °C.

Typical microstructures in the as-cast Al-Cu-Mn-Fe-Si alloys, with different casting conditions, are shown in Figure 3. The as-cast microstructure of studied alloys, consist of α-Al, Al_6_(FeMn), Al_15_(FeMn)_3_(SiCu)_2_ (α-Fe), and Al_2_Cu phases, in accordance with references [19,25]. The gravity casting alloy (Figure 3c) shows coarse dendrite morphology and many shrinkage pores, while the UV+AP processed alloys (Figure 3b,d) shows fine dendrite morphology and pore-free features. Thus, the microstructures processed by UV+AP with different pouring temperatures are effectively refined compared with the gravity casting alloys.

**Figure 4:**
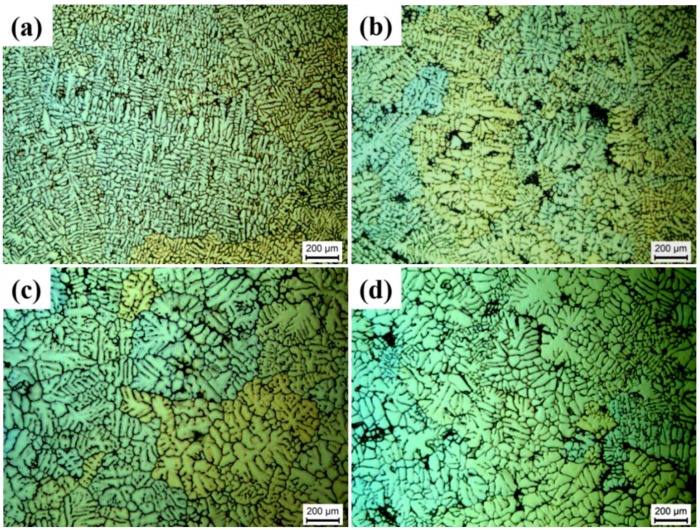
Polarized optical images showing the grain size of the alloys with different pouring temperatures: (**a**) UV+AP, 740 °C; (**b**) UV+AP, 710 °C; (**c**) gravity casting 710 °C; (**d**) UV+AP, 680 °C.

Figure 4 shows the polarized optical images of the grain size in the as-cast alloys processed by different pouring temperatures. The primary α-Al phases in the gravity casting alloy (Figure 4c) shows the coarse dendritic morphology. The primary α-Al phases in the UV+AP processed alloys (Figure 4a,b) are significantly finer than the gravity casting alloy (Figure 4c). The grain sizes of primary α-Al phases in the UV+AP state with the pouring temperatures of 740, 710, and 680 °C are 803 ± 194 μm, 417 ± 78 μm, and 495 ± 108 μm, while 634 ± 211 μm is in the gravity casting alloy. The values of secondary dendrite arm spacing (SDAS) of individual alloys showed a similar trend. The microstructure analysis confirmed that the alloy processed by UV+AP, with a pouring temperature of 710 °C, has the smallest grain size. This is due to the higher cooling rate resulting in the fine grain size and will be discussed later.

### 3.2. 3D Microstructure

**Figure 5:**
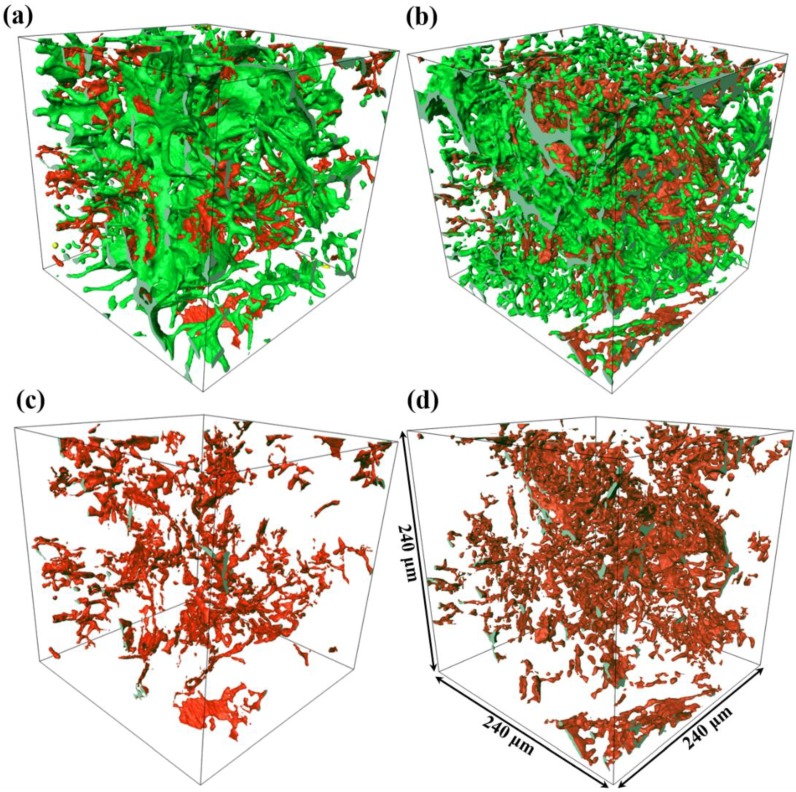
The 3D visualization of the intermetallic phases for different casting conditions: (**a**,**c**) gravity casting; (**b**,**d**) processed by UV+AP with the pouring temperature of 710 °C. Red color represents Fe-rich phases, green color represents Al_2_Cu.

Figure 5 shows the visualizations of the 3D networks formed by intermetallic phases with different casting conditions in a volume of 240 × 240 × 240 μm^3^. The alloys with gravity casting and processed by UV+AP with the pouring temperatures of 710 °C are comparatively studied. The segmented microstructure is consisted of Fe-rich phases (red color) and Al_2_Cu (green color). As shown in Figure 5a,b, the 3D morphologies of Fe-rich phases and Al_2_Cu in both alloys are interconnected network structure. These intermetallic phases are mainly located in the interdendrite region because they are formed through the eutectic reaction at later stage of solidification. In order to clearly show the 3D morphology of Fe-rich phases, the individual 3D structures are shown in Figure 5c,d. It is clearly shown that the 3D structures of Fe-rich phases in the UV+AP are more compacted than those in the gravity casting alloy.

**Figure 6:**
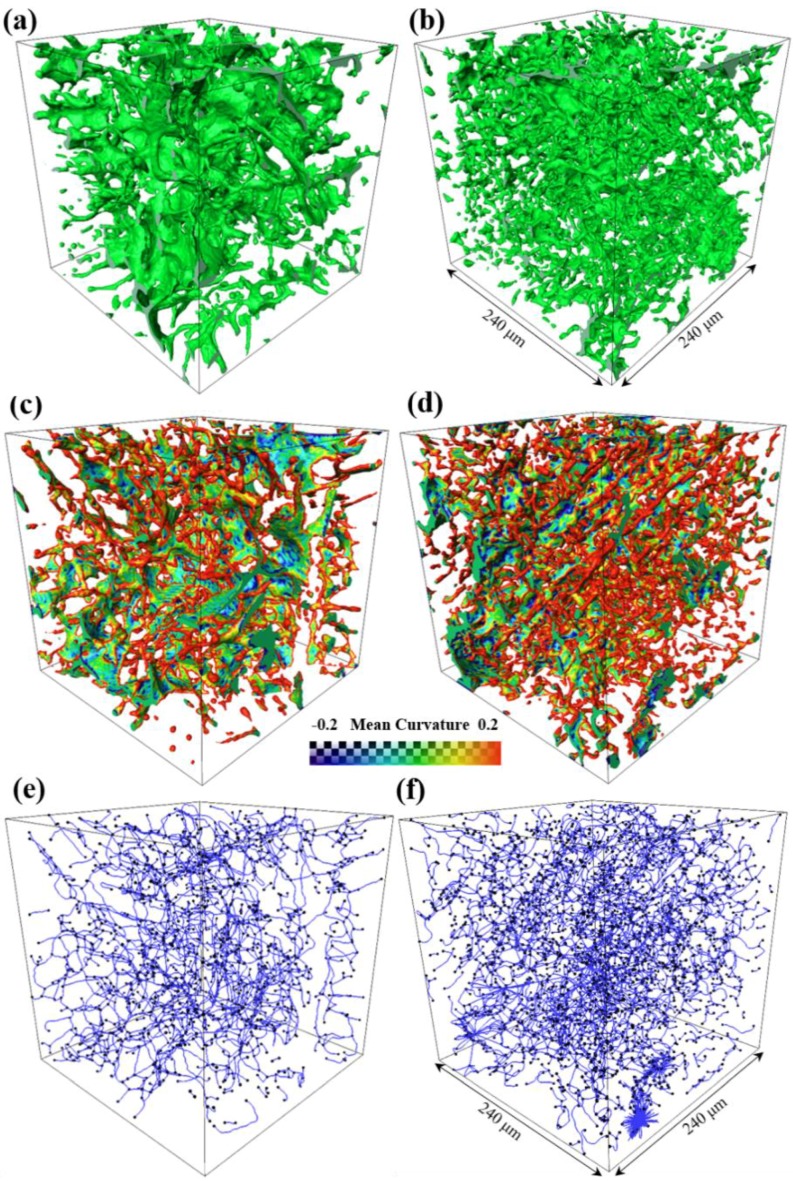
The 3D visualization of the Al_2_Cu for different casting conditions: (**a**,**c**,**e**) gravity casting; (**b**,**d**,**f**) processed by UV+AP with the pouring temperature of 710 °C. (**a**,**b**) 3D morphology; (**c**,**d**) mean curvature; (**e**,**f**) skeletonization analysis.

Volume rendered Al_2_Cu particles and their mean curvature and skeletonization analysis on different casting conditions are shown in Figure 6. It is found that the morphology of Al_2_Cu particles processed by UV+AP is much finer than those in gravity casting, as shown in Figure 6a,b. Figure 6c,d present the mean curvatures of 3D morphology of Al_2_Cu particles for the UV+AP, and gravity casting alloy, respectively. The Al_2_Cu particles in the UV+AP alloy have more positive and negative mean curvatures. The skeletonization analysis (Figure 6e,f) of both alloys show that the Al_2_Cu particles in the UV+AP alloy have more branches and are more compacted.

Table 2 indicates the volume and skeletonization analysis of Al_2_Cu in a volume of 240 × 240 × 240 μm^3^. The total volumes are 121,712 and 98,209 μm^3^, respectively, and their volume fractions are 0.88%, and 0.71%, respectively. Moreover, the segmented numbers, mean length, and mean radius are relatively reduced while the node number is increased. This indicates that the 3D morphologies of Al_2_Cu particles are remained highly interconnected after UV+AP processed, while they are become more compacted and refined.

**Figure 7:**
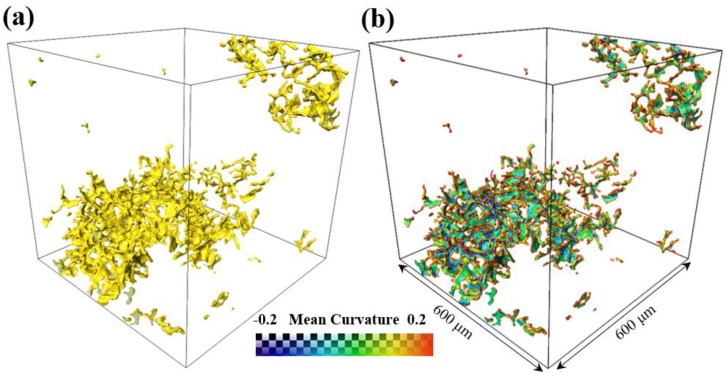
The 3D morphology of the pores in the gravity casting alloy: (**a**) 3D structure; (**b**) mean curvature.

Figure 7 shows the 3D reconstructed images of pores in a volume of 600 × 600 × 600 μm^3^ in the gravity casting alloy. As shown in Figure 7a, the highly curved and interconnected pores are randomly distributed in the alloy. This arises as a result of lack of liquid melt feeding during solidification. However, nearly no pores exist in the alloys processed by UV+AP (so the figure is not shown here). The mean curvatures of pores in the alloy (Figure 7b) show that the protrusion and depression region are highly limited by the primary Al dendrite and are located in the interdendrite region [35].

### 3.3. Heat Treatment Microstructural Analysis

**Figure 8:**
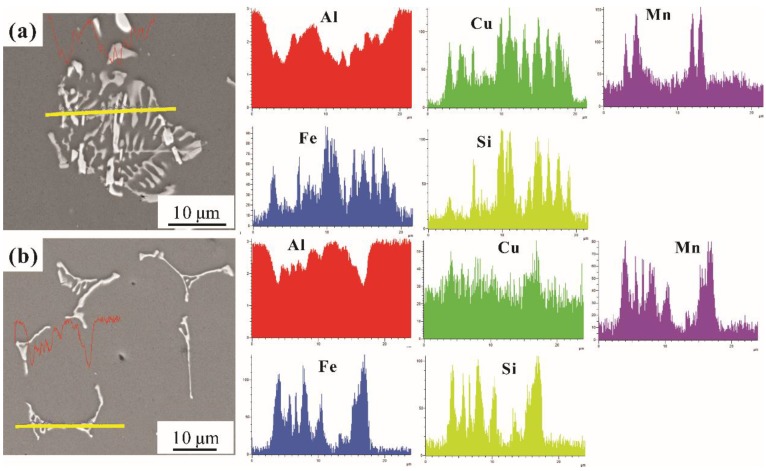
Line scanning of Fe-rich phases: (**a**) α-Fe and α(CuFe) phases in the gravity casting alloy; (**b**) α-Fe in the alloy processed by UV+AP with the melting temperature of 710 °C.

Figure 8 shows the 2D morphology of T5 heat-treatment intermetallic phases with SEM backscatter mode. From Figure 8a,b, the line scanning of Fe-rich phases in the examined alloys are identified by EDS. The detailed compositions are listed in Table 3. With the aid of EDS, the Cu content in the white phase is up to 13.1%, while the Mn and Si content are both 1.1%. Thus, the white phase is identified as α(CuFe) (Al_7_Cu_2_Fe), and the skeleton-like grey phase is determined as α-Fe (Al_15_(FeMn)_3_(SiCu)_2_). The results are in good agreement with the literature [6].

**Figure 9:**
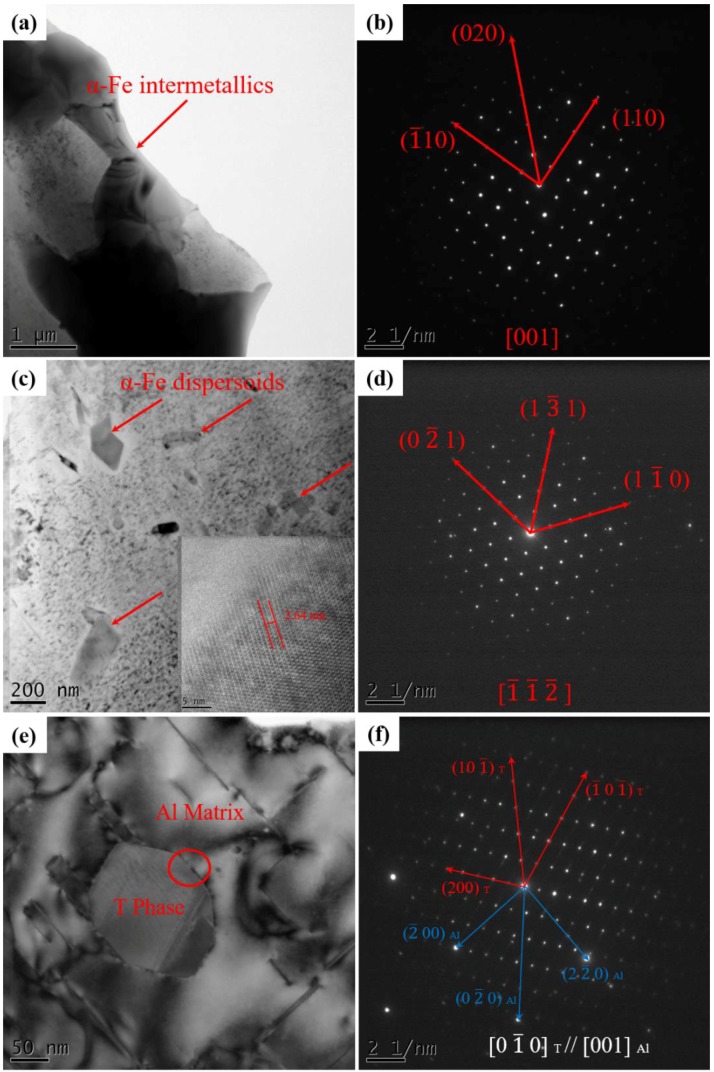
Identification of intermetallic phases and dispersoids: (**a**,**b**) α-Fe intermetallic phases; (**c**,**d**) α-Fe dispersoids; (**e**,**f**) T phase and Al matrix.

Figure 9 shows the TEM micrographs of intermetallic phases and dispersoids in alloys after T5 heat treatment. As shown in Figure 9a,c, the Chinese-script intermetallic phases and block-like dispersoids are present in the alloys. Figure 9b,d show the selected area electron diffraction patterns (SADPs) of the corresponding particles. Combing with SADP and EDS, the Chinese-script particle is identified as α-Fe, and has a body-centered cubic (BCC) unit cell of *a* = *b* = *c* = 1.265 nm. Figure 9d shows SADPs of single dispersoids alone [$\bar{1}\bar{1}\bar{2}$] zone axes. The dispersoids have a different crystal structure with α-Fe. The dispersoids have an single cubic structure with a lattice *a* = 1.32 nm, which is in accordance with the single cubic α-Al(MnFe)Si (α-Fe) phases [40]. The HRTEM image inserted in Figure 9c indicate that the distance between 2 raw of atoms is 2.64 nm, which further confirm the existence of α-Fe phase. As shown in Figure 9e, the block-like dispersoids are distributed in the matrix. The SADPs confirm that the single dispersoid is Al_20_Cu_3_Mn_2_ (T) phase, which has a B-centred orthorhombic structure with *a* = 2.420 nm, *b* = 1.250 nm, *c* = 0.772 nm, space group of *Bbmm*. The SADPs of single T dispersoids indicates that [$0\bar{1}0$]_T_ zone axes is parallel to [001]_Al_.

**Figure 10:**
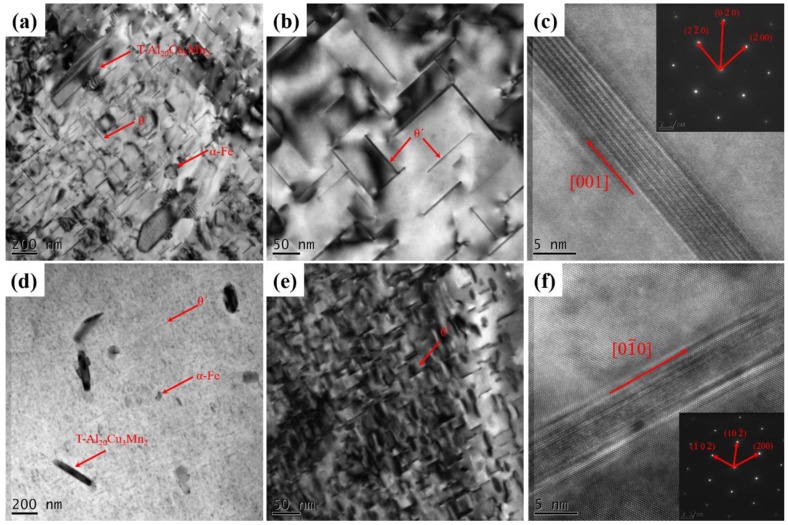
Dispersoids in the alloys processed by different casting condition: (**a**–**c**) gravity casting; (**d**–**f**) processed by UV+AP with the pouring temperature of 710 °C.

In order to study the effect of UV+AP on the sizes and morphology dispersoids in the alloys, the bright-field TEM images are shown in Figure 10. As can been seen from Figure 10a,c,e, there are three kinds of dispersoids in the alloys: α-Fe, T and θ’. It is shown that the UV+AP lead to a significant size reduction of the three dispersoids. As presented in Figure 10 b,c,e,f, the plate-like phases are identified by the SDAPs as θ’ phase and their observation directions are along <001> direction. It was also established that the size and length of θ’ phase after UV+AP processed are significantly reduced. The length and thickness of θ’ phase in the Al matrix have been determined by the TEM images. Their length and thickness in the gravity casting alloy are 110 ± 29 nm and 3.4 ± 1.1 nm, respectively. While, the sizes are decreased to 43 ± 21 nm, and 1.7 ± 0.8 nm after UV+AP processed, respectively.

### 3.4. Tensile Test

**Figure 11:**
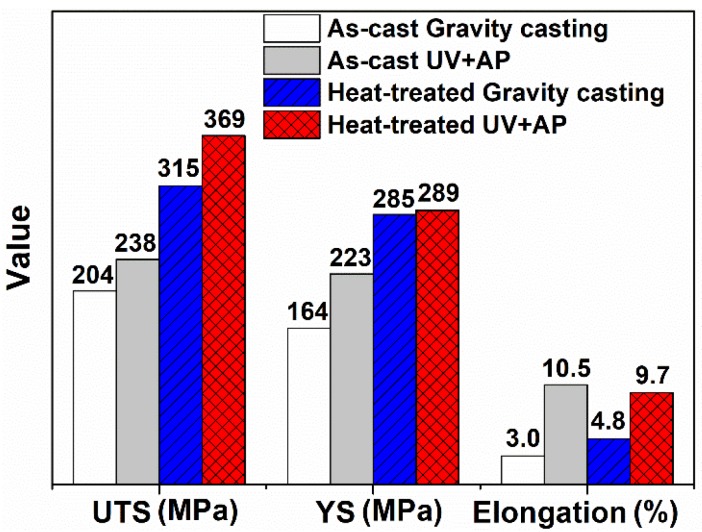
The mechanical properties of the alloys under different casting conditions.

Figure 11 shows the tensile strength and elongation of as-cast and T5 heat-treatment of the alloys under the different casting conditions. Compared with the alloy under gravity casting, the values of ultimate tensile strength (UTS), yield strength (YS), and elongation of alloys under UV+AP increased. In the T5 heat-treatment condition, when the processing is changed from gravity casting to UV+AP, the values of UTS are increased from 315 MPa to 369 MPa, the values of YS are increased from 285 MPa to 289 MPa and the values of elongation are increased from 4.8% to 9.7%. The values of UTS and YS of the heat-treatment alloys are higher than those of in as-cast state, while the elongation value is relatively reduced. This is due to the Fe-rich phases become spheroidized following heat treatment.

**Figure 12:**
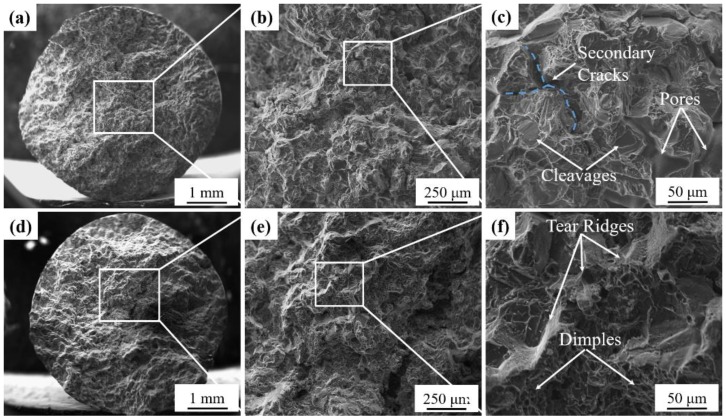
The fracture surfaces of the alloys under different casting conditions: (**a**–**c**) gravity casting; (**d**–**f**) UV+AP.

The fracture surfaces of the alloys in the both alloys under gravity casting and UV+AP in heat-treatment state are presented Figure 12. Figure 12a–c exhibits the typical fracture surfaces morphologies of the shrinkage pores, secondary cracks, and cleavages in the gravity casting alloy. It can be clearly seen that the fine dimples and tear ridges are uniformly distributed in the alloys, as shown by the white arrows in Figure 12d–f. The fine dimples and pore-free features in the alloys indicating that the UV+AP processing significantly improve the elongation of the alloys.

## 4. Discussion

### 4.1. Effect of Pouring Temperature on the Microstructure

**Figure 13:**
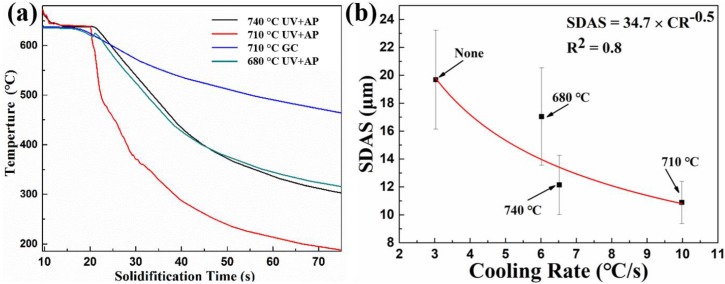
(**a**) Cooling curve for the alloys under different casting conditions; (**b**) the relationship between cooling rate and secondary dendrite arm spacing (SDAS).

The optical microstructures of the alloys under different pouring temperatures are shown in Figure 3 and Figure 4. The coarse dendrites and a small number of pores present in the gravity casting alloy (Figure 3c), while fine dendrites and pore-free microstructure are shown in the UV+AP with the pouring temperature of 710 °C (Figure 4b). The differences in grain size and SDAS for different casting conditions can be deduced by the different cooling rate, which result in different nucleation rates. Figure 13a is the cooling cures for the alloys under different casting conditions. The cooling rates for the alloys under UV+AP with pouring temperature of 680, 710, and 740 °C are 6.52 °C/s, 9.98 °C/s, 6.02 °C/s, respectively. While, the cooling rate for the alloy with gravity casting alloy is 3.03 °C/s. Thus, the UV+AP processing can significantly improve the cooling rate and then influence the grain size. The relationship between cooling rate and SDAS is given by [41]:

SDAS = a × CR^−b^ (2)

The value of b is in the rage of 0.3–0.6. Thus, the calculated formula is:

SDAS = 34.7 × CR^−0.5^ (3)

The relationship between the cooling rate and SDAS for different alloys is plotted in Figure 13b. Our experiment results show good agreement with the empirical formula. The influence of pouring temperature on the evolution of SDAS can also be explained by the solidification theory. As can be seen from Figure 13a, the stage temperatures are formed at the range of 640 °C–650 °C, which is caused by the exothermic reaction during eutectic reaction. The stage temperatures for the alloys with UV+AP of 740 °C and 710 °C are 639 °C–643 °C, while those for the alloys with UV+AP of 680 °C and gravity casting are 635 °C–638 °C. As the pouring temperatures decreased, their undercooling and cooling rate increased. Thus, the alloy with UV+AP of pouring temperature of 710 °C has the largest cooling rate of 9.98 °C/s, resulting in much more nucleates sites for Al grains.

### 4.2. Effect of UV+AP on the 3D Microstructure

The volume rendered Fe-rich phases, Al_2_Cu and pores of the alloys for gravity casting and UV+AP with 710 °C, are presented in Figure 5, Figure 6 and Figure 7. In the alloy with UV+AP, the sizes of intermetallic phases are significantly refined and their morphologies become more compact. This is attributed to the synergistic effect of ultrasonic vibration and applied pressure during solidification. The acoustic cavitation and acoustic streaming effect, generated by ultrasonic sonotrode, involve the main refinement mechanisms [9,10]. As the sonotrode is inserted into the Al melt, the large volume fractions of cavitation bubbles interact with the Al dendrites and intermetallic phases. The implosion of cavitation bubbles lead to the fragmentation of primary dendrite and then intermetallic phases are formed in the narrow channel of interdendrite through the following eutectic reaction. Meantime, the acoustic streaming brings the fragments into the new sites in the melts, which increases the nucleation rate and accelerates the solute atoms diffuses more homogenously. On the other hand, the applied pressure also contributes to the microstructural refinement and enhancement of mechanical properties. It is well-known that the applied pressure can force the melt feeding during solidification and increase the thermal conductivity between the mold and melts. These findings show good agreement with the literature [25,26] by the present authors. Thus, under the UV+AP of combined ultrasonic vibration and applied pressure, both the Al dendrite and intermetallic phases are effectively refined.

### 4.3. Strengthen Mechanism

As shown in Figure 11, the UTS and elongation of the alloy, processed by the UV+AP, are significantly enhanced. The main mechanisms are: Fine-grained strengthening, Orwan strengthening and solution strengthening.

#### 4.3.1. Fine-Grained Strengthening

The grain size of the alloy processed by UV+AP is 417 ± 78 μm, while the value is 634 ± 211 μm for the gravity casting alloy. Thus, the contribution of fine-grained strengthening can be calculated by the Hall-Petch equation,

(4) ∆σg = k (d−12 − d0−12)

where k = 74 MPa μm^1/2^ and *d* and *d*_0_ are the average grain sizes of the UV+AP alloy, and gravity casting alloy, respectively. The strength contribution to ∆$\sigma_{g}$ is calculated to 0.68 MPa. Thus, the grain refinement plays a very small contribution to the improvement of mechanical properties.

#### 4.3.2. Orwan Strengthening

As shown in Figure 9 and Figure 10, the dispersoids of α-Fe, T and θ’ phase are coexisted in the Al matrix. Both contribute to the improvement of the yield strength, and can be calculated by the Ashby equitation [42],

(5) $$\sigma_{Orwan}=\frac{0.8MGb}{2\pi {\left(1-\nu \right)}^{1/2}}\frac{\ln\left(d_{p}/b\right)}{\left(\lambda -d_{p}\right)}$$

where *M* is the Taylor factor, 3.06; *G* is the shear modulus, 27.4 GPa; *b* is burgers vectors of dislocation, 0.286 nm; *v* is the Poisson ratio, 0.33; *d_p_* is diameter of the dispersoids; λ is the interspacing between the two dispersoids, λ=dpπ2fv, which *f_v_* is the volume fraction of dispersoids. Thus, the dispersoids contribution to the Orwan strengthening for the condition of gravity casting and UV+AP are 54 MPa, and 125 MPa, respectively.

#### 4.3.3. Solution Strengthening

As determined by the EDS, the Cu content solid solution in Al matrix in the UV+AP (5.4%) is higher than those of in gravity casting alloy (4.5%). The solid solution strengthening is given by [43],

(6) $$\sigma_{\mathrm{ss}}\;=\;2Ac_{0}^{2/3}$$

where A is the constant, in this study, the value for Cu solubility in Al matrix is 12.4. C_0_ is the concentration of Cu in Al matrix. Owing to the solubility of Fe, Si and Mn in Al matrix is limited, so their contribution to solution strengthening is neglected. Thus, the Cu content contribution to solid solution in Al matrix for the UV+AP and gravity casting alloy is 76 MPa, and 67 MPa, respectively.

## 5. Conclusions

In summary, the effect of compound fields of UV+AP with different pouring temperature of 740, 710 and 680 °C, and gravity casting on recycled Al-Cu-Mn-Fe-Si alloys is comparatively studied. They are systematically studied by means of optical microscopy, scanning electron microscopy, transmission electron microscopy, synchrotron X-ray tomography, and tensile test. After UV+AP treated, the alloy with pouring temperature of 710 °C shows the smallest grain size (417 ± 78 μm) and SDAS. The 3D morphologies of Fe-rich phases and Al_2_Cu are interconnected in networks, and their sizes are greatly reduced after UV+AP treated. The Fe-rich phases in the alloys are changed from α(CuFe) and α-Fe in gravity casting alloy to α-Fe in UV+AP processed alloys. There are three different kind dispersoids: α-Fe, T and θ’ in the UV+AP and gravity casting alloys. When the processed conditions of the alloys are changed from gravity casting to UV+AP, the values of UTS are increased from 315 MPa to 369 MPa, the values of YS are increased from 285 MPa to 289 MPa and the values of elongation are increased from 4.8% to 9.7% in the heat-treatment state. The enhancement of mechanical properties can be explained by the dendrite fragmentation, solute uniformly distribution and microstructural refinement under the synergistic effect of acoustic cavitation, acoustic streaming and forced feeding. The main strengthening mechanisms of Orwan strengthening and solution strengthening are systematically calculated.

## Figures and Tables

**Table 1:** Chemical composition of the studied alloy (wt.%).

Nominal Composition	Cu	Mn	Fe	Si	Al
Al-5.0Cu-0.6Mn-0.5Fe-0.6 Si	5.67	0.65	0.51	0.66	Bal.

**Table 2:** Data analysis of three-dimensional (3D) morphology of Al_2_Cu with different casting conditions.

Casting Conditions	Total Volume (μm^3^)	Number of Segment	Mean Length (μm)	Number of Node	Mean Radius
Gravity casting	121,712	2043	17.38	1723	0.789
UV+AP	98,209	3624	17.21	2926	0.658

**Table 3:** Compositions of the intermetallic compounds in Figure 8 as measured by energy dispersive spectrometer (EDS) (at.%).

Phases	Al	Cu	Mn	Fe	Si	Identified Phases
White	78.7	13.1	1.1	6.0	1.1	α(CuFe) (Al_7_Cu_2_Fe)
Grey	73.6	4.3	4.7	9.7	7.7	α-Fe (Al_15_(FeMn)_3_(SiCu)_2_)
